# Down-regulation of EVA1A by miR-103a-3p promotes hepatocellular carcinoma cells proliferation and migration

**DOI:** 10.1186/s11658-022-00388-8

**Published:** 2022-10-22

**Authors:** Qian Xu, Zhaozhong Liao, Zunshuang Gong, Xiaokun Liu, Yuling Yang, Zhe Wang, Weiyan Yang, Lin Hou, Jiejie Yang, Junying Song, Wenjing Liu, Bin Wang, Junnan Hua, Mingyi Pu, Ning Li

**Affiliations:** 1 Department of Biochemistry and Molecular Biology, School of Basic Medicine, Qingdao University, Qingdao, China; 2 Department of Infectious Diseases, Affiliated Hospital of Qingdao University, Qingdao, China; 3 Department of Biotechnology, School of Basic Medicine, Qingdao University, Qingdao, China; 4 Department of Anesthesiology, Family Planning Service Center, Maternal and Child Health Hospital of Jiaozhou City, Qingdao, China; 5 College of Electronic Information, Micro-Nano Technology College, Qingdao University, Qingdao, China

**Keywords:** EVA1A, miR-103a-3p, hepatocellular carcinoma, Apoptosis, TP53, Autophagy, Mitosis

## Abstract

**Background:**

EVA1A (Eva-1 homolog A), a novel protein involved in autophagy and apoptosis, functions as a tumor suppressor in some human primary cancers, including hepatocellular carcinoma (HCC). While it is consistently downregulated in several cancers, its involvement in hepatocarcinogenesis is still largely unknown.

**Methods:**

We first detected the expression of EVA1A in HCC tissues and cell lines using RT‒qPCR, immunohistochemistry and western blotting and detected the expression of miR-103a-3p by RT‒qPCR. Then, bioinformatics prediction, dual-luciferase reporter gene assays and western blotting were used to screen and identify the upstream microRNA of EVA1A. After manipulating the expression of miR-103a-3p or EVA1A, wound healing, invasion, proliferation, colony formation, apoptosis, autophagy, mitosis and mitochondrial function assays, including mitochondrial membrane potential, ROS and ATP production assays, were performed to investigate the functions of miR-103a-3p targeting EVA1A in HCC cells. Apoptosis-related proteins were assessed by RT‒qPCR (TP53) or western blotting (TP53, BAX, Bcl-2 and caspase-3). Autophagy level was evaluated by observing LC3 puncta and examining the protein levels of p62, Beclin1 and LC3-II/I.

**Results:**

We found that EVA1A expression was decreased while miR-103a-3p expression was increased in HCC tissues and cell lines and that their expression was inversely correlated in HCC patients. The expression of miR-103a-3p was associated with HCC tumor stage and poor prognosis. miR-103a-3p could target EVA1A through direct binding to its 3'-UTR and suppress its expression. Overexpression of miR-103a-3p significantly downregulated the expression of EVA1A, TP53 and BAX, upregulated the JAK2/STAT3 pathway and promoted HCC cell migration, invasion and proliferation, while repression of miR-103a-3p dramatically upregulated the expression of EVA1A, TP53, BAX and cleaved-caspase-3, inhibited HCC cell migration, invasion and proliferation, and caused mitochondrial dysfunction and apoptosis. Overexpression of EVA1A significantly attenuated the cancer-promoting effects of miR-103a-3p in HCC cells, while knockdown of EVA1A alleviated the mitochondrial dysfunction and apoptosis caused by miR-103a-3p inhibition. Overexpression of EVA1A did not induce significant changes in autophagy levels, nor did it affect G2/M transition or mitosis.

**Conclusion:**

These findings indicate that the downregulation of the tumor suppressor EVA1A by miR-103a-3p potentially acts as a key mediator in HCC progression, mainly by inhibiting apoptosis and promoting metastasis. The miR-103a/EVA1A/TP53 axis provides a new potential diagnostic and therapeutic target for HCC treatment.

**Supplementary Information:**

The online version contains supplementary material available at 10.1186/s11658-022-00388-8.

## Background

Hepatocellular carcinoma (HCC), the most common pathological type of primary liver cancer, is the fourth leading cause of cancer-related death worldwide [1]. In recent years, significant progress has been made in the diagnosis and treatment of HCC, but the overall prognosis of patients remains poor, with an overall 5-year survival rate of 10.1% [2]. A large proportion of patients with HCC are diagnosed at a late stage, and recurrence or metastasis of HCC remains common after resection. Therefore, a better understanding of the molecular pathways involved in the etiology and progression of HCC is urgently needed and may lead to improved early diagnosis and treatments.

EVA1A (Eva-1 homolog A), also known as TMEM166/FAM176A, is a novel protein involved in programmed cell death screened by high-throughput in 2007 [3]. As an ER-associated protein, EVA1A can regulate cellular autophagy and apoptosis [4–8]. Studies have shown that EVA1A is expressed in a cell-type-specific and tissue-type-specific manner, and compared with normal tissues, the expression of EVA1A is widely downregulated in tumor tissues [9, 10]. EVA1A has recently been reported to have antitumor activity in several carcinomas and is considered a tumor suppressor gene [5–7]. Our latest research shows that EVA1A expression was significantly decreased in HCC and was associated with advanced TNM clinical stage and poor clinical outcome of HCC patients. Overexpression of EVA1A inhibits HCC growth by upregulating TP53, which makes it possible for EVA1A to be a potential therapeutic target for HCC [10]. However, the regulatory mechanisms of EVA1A expression in HCC remain unclear. One study showed that EVA1A can be downregulated by miR-125b in HCC, thereby increasing the sensitivity of HCC to the chemotherapy drug oxaliplatin, which indicates that the decline in EVA1A expression in the development of HCC may be regulated by miRNAs [11]. The elucidation of the regulatory mechanisms of EVA1A expression in HCC will facilitate the discovery of the underlying mechanisms of HCC tumorigenesis.

The miR-103/107 family has been found to participate in the regulation of many tumors [12]. Studies have shown that miR-103 is abnormally expressed in a variety of cancers. For example, miR-103 is downregulated in non-small cell lung cancer, where it functions as a tumor suppressor gene [13]. On the other hand, miR-103 is upregulated in numerous types of cancers, including neuroblastoma [14], gastric cancer [15], breast cancer [16] and colorectal cancer [17]. More precisely, the regulation of miR-103a-3p has been widely reported in many diseases. For example, miR-103a-3p suppresses cell proliferation and invasion by targeting tumor protein D52 in prostate cancer [18] but promotes human gastric cancer cell proliferation by targeting activating transcription factor 7 (ATF7) in vitro [19], and it also promotes tumor glycolysis through the Hippo pathway in colorectal cancer [20]. In addition, miR-103a-3p was reported to regulate proliferation and apoptosis by targeting RCAN1 in oral squamous cell carcinoma (OSCC) cell lines [21]. Thus, the miR-103/107 family is widely considered an oncogene. Based on these findings, miR-103 may represent a prospective target for both cancer diagnosis and therapy. However, the role of miR-103a-3p in HCC remains unclear.

In this study, we confirmed that EVA1A was downregulated, while miR-103a-3p was notably upregulated in HCC tissues and HCC cell lines, and its high expression was associated with poor prognosis in patients with HCC. We identified that miR-103a-3p downregulates EVA1A by directly targeting its 3ʹ-untranslated region (3ʹ-UTR) to promote HCC cell growth and migration. EVA1A overexpression can significantly reduce the cancer-promoting effects caused by miR-103a-3p mimics. miR-103a-3p repression induces upregulation of EVA1A and TP53, which play an anticancer role by inducing mitochondrial dysfunction and triggering cell apoptosis. Our results indicate that repression of EVA1A by upregulated miR-103a-3p may contribute to HCC development and that the miR-103a-3p/EVA1A/TP53 axis may be a potential signaling mechanism for the tumorigenesis of HCC.

## Material and methods

### Clinical samples

All tissue samples, including HCC tumor samples and matched distant non-cancerous samples, were collected from 25 patients with operable primary HCC who underwent surgery in 2021 at the Affiliated Hospital of Qingdao University. Informed consent was obtained from each patient, and the ethics committee of Medical College of the of Qingdao University approved the study. For analysis the correlation of the expression of EVA1A with the clinicopathological features of HCC patients, HCC tissue microarrays, containing samples from more than 900 patients, were purchased from SHANGHAI OUTDO BIOTECH Company and subjected to EVA1A IHC analyses according to previous study[10].

### Haematoxylin and eosin (H & E) staining assay

Fresh tissues were immobilized immediately after liver resection, paraffin-embedded and sliced. Then, the samples were stained with hematoxylin for 5 min, washed with running water for 5 min, soaked in xylene and alcohol, dyed with 0.5% eosin for 3 min and re-immersed in alcohol and xylene. Specimens were sealed using a synthetic resin.

### Immunohistochemistry (IHC)

Tissue sections were deparaffinized, rehydrated and incubated in 0.01 M citrate buffer (pH 6.0) for 30 min at 95 °C. Then, the sections were washed 3 times in PBS. Soak in 3% H_2_O_2_ for 10 min to inhibit endogenous peroxidase activity. Next, block with ready-to-use goat serum (AR0009) BOSTER for 1 h. Afterwards, the sections were incubated with TMEM166 (Abeam, UK) antibody overnight at 4 °C, followed by incubation with secondary antibody for 2 h at room temperature, and after 3 washes with PBS, were stained by using 3,3-diaminobenzidine (DAB). The original body was observed for immunoreactivity. Sections were stained with hematoxylin and examined under a microscope.were stained with hematoxylin and examined under a microscope.

### EVA1A siRNAs synthesis

Double-stranded siRNAs against EVA1A (si-EVA1A: sense 5ʹ- UGAUAAGGAUCUCUUGCCATT-3ʹ; antisense 5ʹ-UGGCAAGAGAUCCUUAUCATT-3ʹ) were designed chemically synthesized by GenePharma Corporation (Shanghai, China). The control siRNA had no sequence homology to any known human genes. The transfection of siRNA was performed by using Lipofectamine 2000 reagent (Invitrogen, Carlsbad, CA, USA).

### Cell culture and transfection

The immortalized normal human liver cell line L02 and HCC cell lines Hccl-M3, QGY-7703 were obtained from our laboratory. HCC cell line PLC-PRF5 was kindly provided by Dr. Yingyu Chen (Peking University, Beijing). All cells were routinely grown in DMEM (HyClone, USA) with 10% FBS (BI, USA) at 37℃ in a humidified chamber under an atmosphere of 5% CO_2_. Transfection was performed using Lipofectamine 2000 according to the manufacturer’s protocol. 6 h after transfection, cells were cultured in normal medium. Analysis was performed within 72 h of micro-RNA transfection and 24 h after EVA1A transfection.

### Semi-quantitative RT-PCR and and qRT-PCR analysis

Total RNA was extracted from tissue samples and HCC cell lines using Trizol (TaKaRa, Japan) according to the manufacturer’s instructions. Complementary DNA was synthesized using the cDNA Reverse Transcription Kit (HiScript® II Q RT superMix, Vazyme, China), and PCR was performed with 10 pmol of primers and MonAnp^TM^2 × Taq Mix Pro (Monad). The expression of mRNA was also analyzed by quantitative real-time PCR with SYBR Green Master Mix (ChamQTM SYBR® Color qPCR Master Mix, Vazyme, China). Real‐time PCR was performed with a CFX96 Touch Real‐Time PCR Detection System (Bio‐Rad, Hercules, CA, USA). The relative miR-103a-3p and EVA1A mRNA samples was normalized to that of U6 and glyceraldehyde-3-phosphate dehydrogenase (GAPDH), respectively. The primers used are listed below.

miR-103a-3p-forward, 5ʹ-GCGAGCAGCATTGTACAGGG-3ʹ.

miR-103a-3p- reverse, 5ʹ-AGTGCAGGGTCCGAGGTATT-3ʹ.

U6-forward, 5ʹ-AAAGCAAATCATCGGACGACC-3ʹ.

U6- reverse, 5ʹ-GTACAACACATTGTTTCCTCGGA-3ʹ.

EVA1A-forward, 5ʹ-AGATGGCTTTGCTCAGCAACA-3ʹ.

EVA1A-reverse, 5ʹ-GATGCACACGCCAGAAACAA-3ʹ.

EVA1A-AS-forward, 5ʹ-CCTGCATCACTGCATTTCCG-3ʹ.

EVA1A-AS-reverse, 5ʹ-TGCGAAAGAGTGGCACACAG-3ʹ.

TP53-forward, 5ʹ-GAGAGCTGAATGAGGCCTTG-3ʹ.

TP53-reverse, 5ʹ-TTATGGCGGGAGGTAGACTG-3ʹ.

GAPDH-forward, 5ʹ-AACGGATTTGGTCGTATTGGG-3ʹ.

GAPDH-reverse, 5ʹ-TCGCTCCTGGAAGATGGTGAT-3ʹ.

### Protein preparation and western blotting

Tissue samples and HCC cells were lysed using RIPA lysis buffer supplemented with PMSF (1:100, G-CLONE) to obtain total proteins. The proteins concentration was estimated using the BCA protein quantification kit (Solarbio). Equal amounts of protein were subjected to SDS-PAGE, proteins were then transferred onto PVDF membranes (PerkinElmer). After blocking with skimmed milk or BSA, the membranes were incubated with the primary antibodies overnight at 4 ℃. Primary antibodies were as follows: anti-EVA1A (1:500, Abcam), anti-TP53 (1:1000, OriGene), anti-BAX(1:2000, OriGene), anti-BCL-2 (1:2000, Bioss), anti-p-JAK2 (1:1000, Cell Signaling Technology), anti-p-STAT3 (1:1000, Cell Signaling Technology), anti-MMP-9 (1:1000, Cell Signaling Technology), anti-LC3 (1:1000, MBL), anti-p62 (1:1000, Proteintech), anti-Beclin1 (1:2000, Proteintech) and anti-β-actin (1:1000, Servicebio). And then the blot was incubated with peroxidase-conjugated sheep anti-rabbit IgG (1:3000, Servicebio). Protein bands were detected using the ECL system. Each independent experiment was performed at least three times.

### Luciferase reporter assay

The EVA1A 3′-UTR (untranslated region) and mutant luciferase plasmids were obtained from Hanbio (Shanghai, China). HEK293T cells were co-transfected with miR-103a-3p mimics or mimic controls and wild-type or mutated EVA1A-3′UTR plasmids. 48 h after transfection, luciferase activity was measured using the Dual-Luciferase Assay System (Promega) according to the manufacturer’s instructions.

### Cell proliferation assay

The proliferation of cells was measured by Cell Counting Kit (CCK-8) assay. Cells were seeded in a 96-well plate with 100 µL medium per well containing 2000 cells totally, then cells were conventionally incubated for 1 to 4 days at 37 ℃ in a carbon dioxide incubator. At the indicated times, 10 µL CCK8 reagent was added to each well and incubated at 37 ℃ for 4 h. The absorbance of the samples was measured at 450 nm by plate reader (Bio-Rad Laboratories). Each independent experiment was run in triplicate.

### Wound healing assay

The migratory capacity of tumor cells was measured by wound healing assay. Cells were seeded in 6-well plates, 24 h after transfection, straight wounds were generated using a 200 µL sterile pipette tip. Then the floating cell debris was rinsed off by washing with PBS and readding serum free DMEM. The cells were conventionally incubated for 1 to 2 days at 37 ℃. Photographs were taken at different time points (0, 12, 24 and 48 h) to assess the wound healing area. The open wound area was measured using Image J software.

### Transwell matrigel invasion assay

Transwell chambers (Corning, NY, USA) had a base membrane pore size of 8 μm, and the chambers were coated with Matrigel (Sigma) to determine invasive capacity. A cell suspension of 1 × 10^5^ cells/mL was prepared, 200 µL of the suspension was inoculated into each upper chamber with serum-free medium. 12 h later, 600 µL of culture medium containing 10% fetal bovine serum was added to the lower chamber. After 24 h of incubation in 37 °C, the non-migrating cells on the top chamber were completely removed with a cotton swab. Cells that migrated to the bottom chamber were then fixed with methanol for 30 min, stained with 0.1% crystal violet for 15 min, and cells were counted and visualized in five random fields under a microscope.

### Colony formation assays

Long-term survival of transfected cells was examined using a plate colony formation assay. Cells were plated into 6-well plates (2000 cells/well) and cultured at 37 ℃ under an atmosphere containing 5% CO_2_. 14 days later, cells were fixed by methyl alcohol for 20 min, and stained with 0.1% crystal violet for 20 min. Then colonies were counted and photographed. All assays were independently performed in triplicate.

### Annexin V/PI apoptosis analysis

Cell apoptosis rate was evaluated by the Annexin V-FITC/PI Apoptosis Detection Kit (BD, USA). Cells were harvested using trypsin without EDTA, washed twice with cold PBS, washed one time with cold binding buffer, and resuspended in 200 µL cold binding buffer. Then the cells were incubated with Annexin V-FITC staining solution and PI staining solution for 15 min at room temperature in the dark. Finally, the samples were immediately analyzed on a flow cytometer (Becton Dickinson, USA). Three independent experiments were performed.

### Detection of mitochondrial membrane potential

Mitochondrial membrane potential was determined using the JC-1 assay kit (Solarbio, CN). The decrease of mitochondrial membrane potential is a landmark event in the early stage of apoptosis. The JC-1 dye aggregates in the mitochondria of healthy cells and emits red fluorescence. However, in unhealthy cells, due to the drop or loss of mitochondrial membrane potential, the JC-1 dye cannot aggregate in the mitochondria, and remains as monomers in the cytoplasm and emits green fluorescence. The transition of fluorescence is usually used as an indicator of early apoptosis. 72 h after transfection, cells were washed with PBS, stained with JC-1 for 20 min, observed under the fluorescence microscope (Nikon, Japan).

### Determination of reactive oxygen species (ROS)

Intracellular ROS levels were determined by the non-fluorescent probe 2, 7-dichlorofluorescein diacetate (DCFH-DA) (Beyotime Biotechnology, CN). DCFH-DA can passively diffuse into cells and be hydrolyzed by esterase to form DCFH. In the presence of ROS, ROS reacts with DCFH to produce fluorescent DCF. Cells were washed two times with culture medium without serum, then incubated with 1 ml culture medium without serum, added DCFH-DA at a final concentration of 10 µM and incubated for 20 min at 37 ℃ in a humidified chamber. Then DCF fluorescence intensity was detected by fluorescence microscope (Nikon, Japan) at excitation wavelength 488 nm and at emission wavelength 525 nm.

### Determination of ATP content

Intracellular ATP levels were determined by the ATP Assay Kit (Beyotime Biotechnology, CN) according to the manufacturer's instructions. In brief, ATP detection working buffer (100 µL) was gently mixed with the cell lysate substrate, then the luminescence was measured using a micro-plate reader (Bio-Rad Laboratories).

### Immunofluorescence staining

Cells were seeded in 24-well plate with cell slides and cultured overnight. After transfection for 24 h, cells were washed with PBS, fixed with 4% paraformaldehyde for 15 min, permeabilized with PBS containing 0.1% Triton X-100 for 20 min, and blocked with ready-to-use goat serum (BOSTER, CN) for 1 h. According to the method in a recent report [22], cells were incubated with appropriate primary antibodies (for example, anti-β-Tubulin (1:200, affinity) overnight at 4 °C and then incubated with secondary antibodies (Alexa Fluor 594-conjugated Goat anti-Mouse IgG (#AS054, Abclonal)) for 1 h at room temperature. DAPI reagent was used to stain cell nuclei. Data were visualized and analyzed with confocal microscopy (STELLARIS 5, Leica) with a 63 × Plan Apochromat 1.4 NA objective.

### Statistical analysis

All experiments were conducted for at least three independent times. Data are expressed as the mean ± standard deviation (SD). GraphPad Prism 6 (USA) was used for all statistical analyses, Comparisons were performed using a student’s t test or 1-way ANOVA, and differences were considered statistically significant at *P* < 0.05.

## Results

### The expression of EVA1A is decreased in HCC tissues and HCC cell lines

**Fig. 1:**
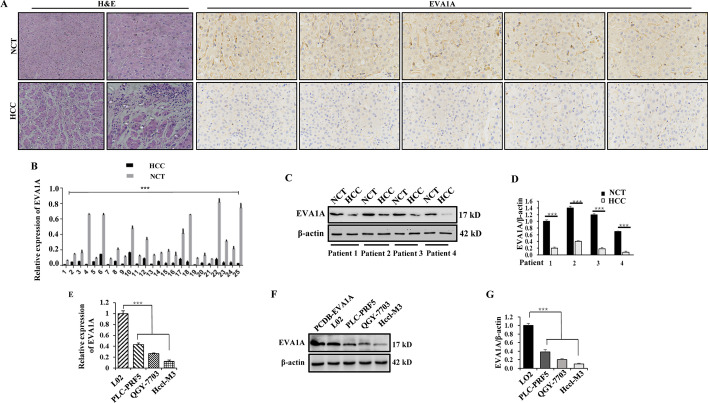
EVA1A protein and mRNA levels in HCC tissues and HCC cell lines. **A** IHC staining of TMEM166 expression in HCC tissues and paired distant non-cancerous tissues (NCT) distinguished with H&E staining. **B** RT-qPCR analysis of EVA1A mRNA levels in twenty-five pairs of HCC and NCT samples. **C**, **D** Western blotting analysis of the expression levels of the EVA1A protein in HCC and NCT samples and quantitative analysis of the blot bands of EVA1A with ImageJ v1.53 k. β-actin was used as loading control. **E**, **F**, **G** RT-qPCR analysis of EVA1A mRNA levels (**E**) and western blotting analysis of EVA1A protein levels (**F**, **G**) in the normal human hepatocyte L02 cells and HCC cell lines. PCDB-EVA1A, an unlabeled plasmid, was used as a positive control. Data are presented as means ± SD of triplicate experiments. ***P < 0.001

We collected 25 pairs of HCC tumor tissues and the corresponding adjacent noncancerous tissues and determined the expression of EVA1A by immunohistochemical staining, RT‒qPCR and western blotting. H&E staining and immunohistochemistry results clearly showed that EVA1A expression was significantly lower in HCC tissues than in matched adjacent nontumor tissues (Fig. 1A). In all 25 HCC samples, the EVA1A mRNA levels were significantly lower than those in their normal adjacent tissue pairs (*P* < 0.001; Fig. 1B). Consistent with the IHC results, western blot analysis showed that the EVA1A protein level in HCC tissues was markedly decreased (*P* < 0.001; Fig. 1C, D), confirming observations we made previously [10]. We further assessed EVA1A expression levels in HCC cell lines and obtained consistent results with HCC tissues. EVA1A mRNA levels and protein levels in PLC-PRF5, Hccl-M3, and QGY-7703 cells were significantly lower than those in the immortalized normal human liver cell line L02 (*P* < 0.001, Fig. 1E–G).

### EVA1A is a direct target of miR-103a-3p

**Fig. 2:**
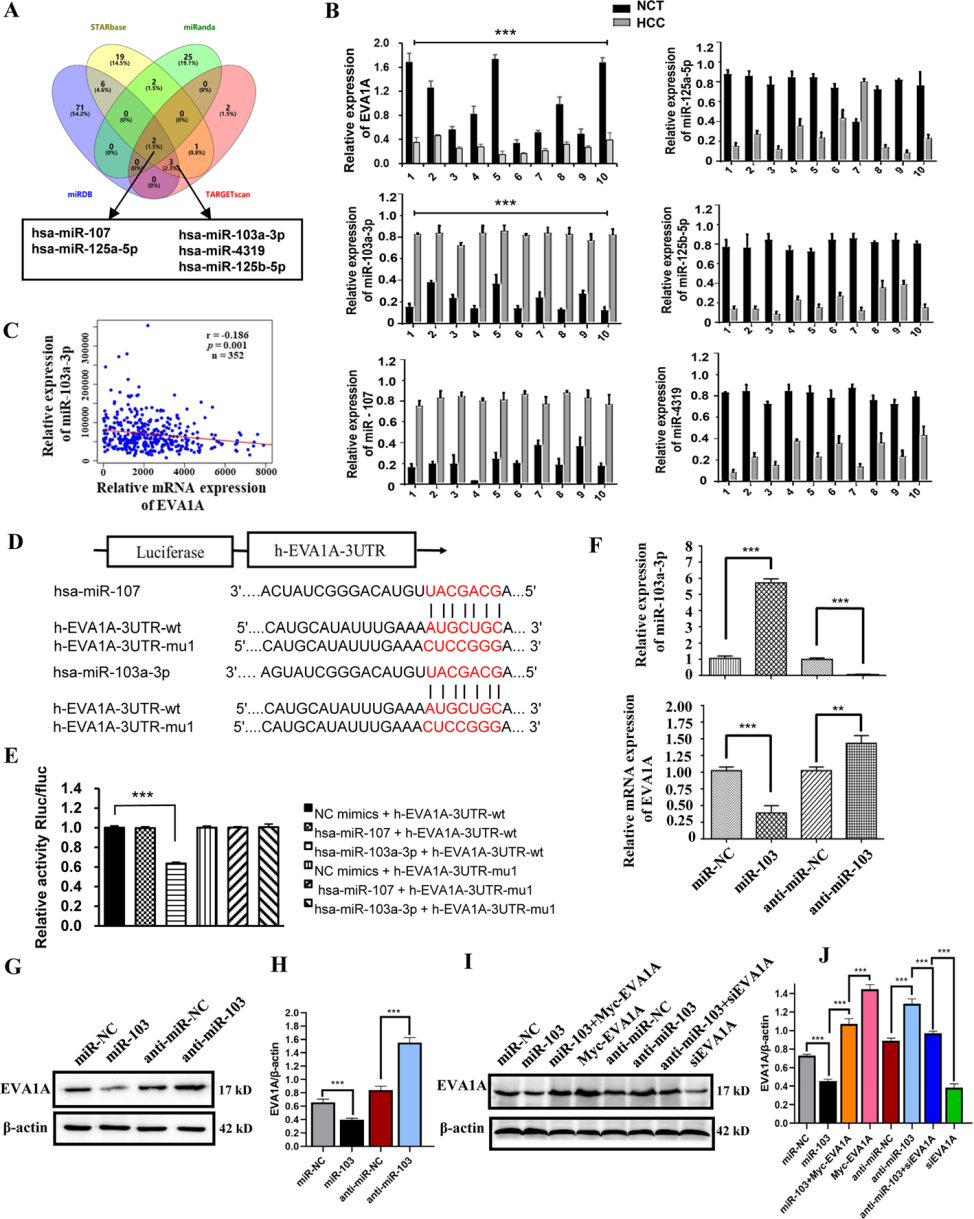
EVA1A is a direct target gene of miR-103a-3p. **A** The potential upstream microRNA of EVA1A by microRNA prediction databases. **B** Ten pairs of samples were randomly selected, RT-qPCR analysis of EVA1A mRNA levels and predicted five microRNA levels. **C** miR-103a-3p expression was inversely correlated with EVA1A levels according to data from HCC patients in the TCGA cohort (*p* = 0.001; Pearson correlation). **D** Scheme of the potential binding sequence of miR-103a-3p and miR-107 within the human EVA1A 3′UTR and construction wild-type (wt) and mutated (mut) luciferase plasmids basing on the EVA1A 3′UTR binding sequence. **E** Human miR-103a-3p or mimic controls (NC mimics) were co-transfected wt or mut EVA1A-3′UTR in 293 T cells and luciferase assay was conducted. **F** Hccl-M3 cells were transfected with synthetic mimics of miR-103a-3p (miR-103) or its control miR-NC, inhibitors of miR-103a-3p (anti-miR-103) or its control anti-miR-NC, then the relative expression of miR-103a-3p and EVA1A was determined by RT-qPCR. **G**, **H** Hccl-M3 cells were treated as indicated, and the EVA1A protein level was determined by western blot. **I** Hccl-M3 cells were co-transfected with miR-103a-3p mimics and Myc-EVA1A plasmid, or were co-transfected with miR-103a-3p inhibitors and siRNA of EVA1A (siEVA1A), or transfected with Myc-EVA1A plasmid or siEVA1A. Then the EVA1A protein level was determined by western blot. **J** Quantitative analysis of the blot bands of EVA1A with ImageJ v1.53 k. Data are shown as means ± SD from three independent experiments. **P < 0.01, ***P < 0.001

Previously, we reported that low expression of EVA1A is associated with the progression of HCC and might be a potential biomarker for poor prognosis of HCC. However, the regulation of EVA1A expression in HCC is not clear thus far. miRNA has been found to be abnormally expressed in HCC, whose central role is gene-expression regulation and some of which are involved in the progression of cancer. We explored the possibility that the differential EVA1A expression in HCC is regulated by miRNA. To identify the potential upstream miRNAs of EVA1A, we searched four databases, miRDB, TargetScan, STARbase and miRanda, and five miRNAs (miR-107, miR-103a-3p, miR-125a-5p, miR-125b-5p, miR-4319) were identified as candidate regulators of EVA1A (Fig. 2A). Next, we performed RT‒qPCR to analyze the expression levels of EVA1A and the five candidate miRNAs in 10 randomly selected pairs of clinical samples. The results showed that in HCC tissues, EVA1A was significantly downregulated, miR-107 and miR-103a-3p were significantly upregulated, while the other 3 miRNAs were downregulated (Fig. 2B), which was excluded according to the concept that miRNAs should have expression patterns that are opposite to those of their targets [23, 24]^.^

In addition, the prediction results demonstrated that the 3′-UTR of EVA1A contains putative complementary binding sites for miR-103a-3p and miR-107 (Fig. 2D). To identify which one could target EVA1A, HEK293T cells were transfected with miR-103a-3p or miR-107 mimics and the luciferase reporter vector harbouring the wild-type or mutant 3ʹ-UTR of EVA1A, respectively. The results demonstrated that overexpression of miR-103a-3p significantly decreased the luciferase activity of cells expressing the wild-type but not the mutant 3′-UTR of EVA1A (Fig. 2E). Furthermore, upregulation of miR-103a-3p with synthetic miR-103a-3p mimics reduced the mRNA expression of EVA1A in Hccl-M3 cells (*P* < 0.001, Fig. 2F). In contrast, downregulation of miR-103a-3p with miR-103a-3p inhibitors had the opposite effect on the mRNA level of EVA1A (*P* < 0.01; Fig. 2F). Together with an analysis using the TCGA database, showing that miR-103a-3p had significant negative correlations with EVA1A (*p* = 0.001, r =  − 0.186; Fig. 2C), it was confirmed that miR-103a-3p interacts with the EVA1A-3′UTR and is able to inhibit its promoter activity. In addition, western blot results showed that miR-103a-3p overexpression led to an obvious decline in the EVA1A protein level, whereas miR-103a-3p inhibition had the opposite effect on EVA1A expression in Hccl-M3 cells (*P* < 0.001; Fig. 2G, H). Another restoration experiment in which miR-103a-3p mimics/inhibitor were co-transfected with Myc-EVA1A plasmids or EVA1A siRNA in Hccl-M3 cells showed that exogenous Myc-EVA1A expression attenuated the decline in the EVA1A protein level induced by miR-103a-3p overexpression, and si-EVA1A counteracted the increase in the EVA1A protein level caused by inhibition of miR-103a-3p (*P* < 0.001; Fig. 2I, J). The above data indicated that *Eva1a* is one target gene of miR-103a-3p and that miR-103a-3p may regulate EVA1A expression both by mRNA degradation and by translational repression.

### Expression of miR-103a-3p and its association with prognosis in patients with liver cancer

**Fig. 3:**
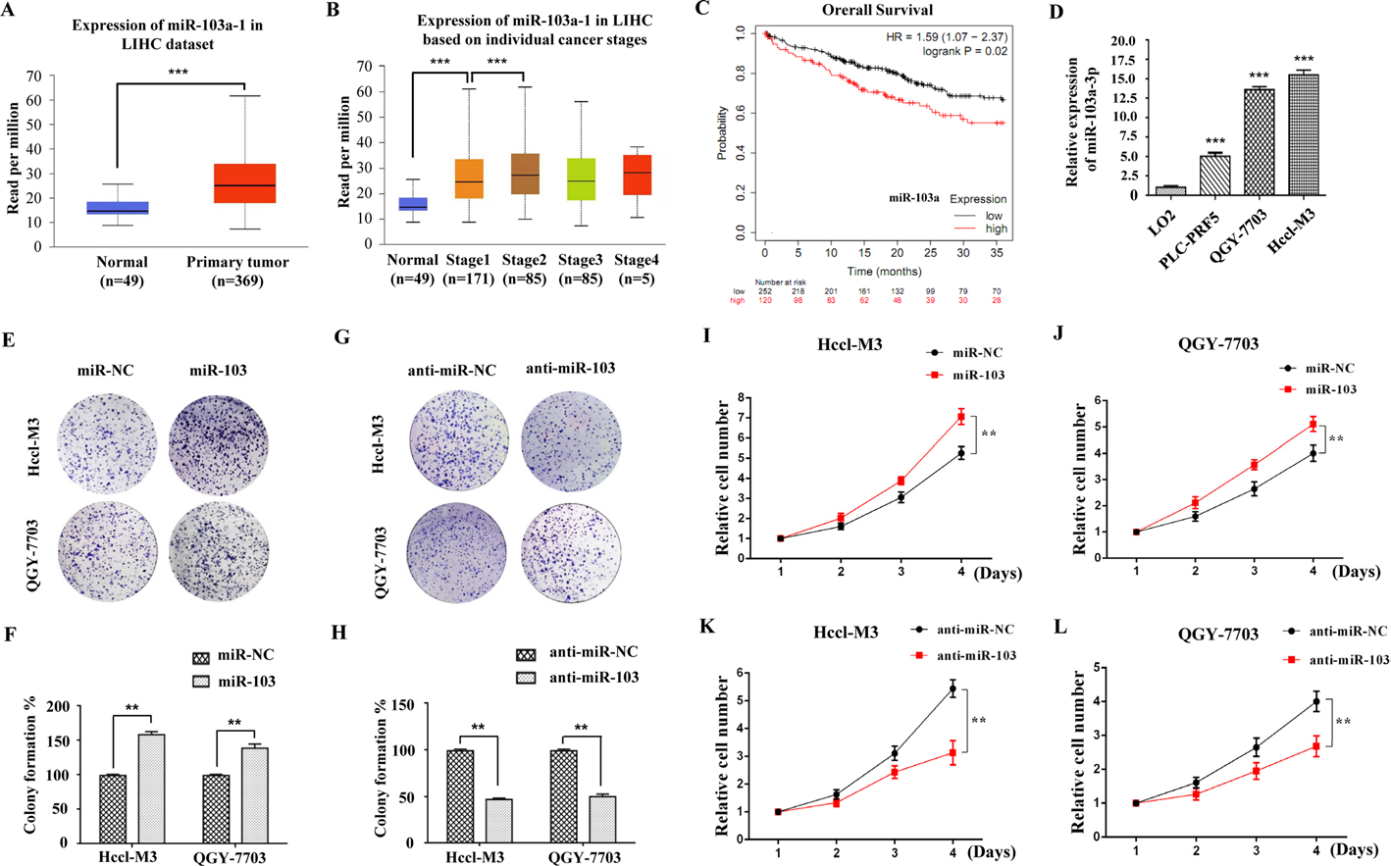
The prognostic significance of miR‑103a in patients with HCC and miR-103a-3p promotes HCC cells proliferation. **A** Expression of miR-103a-3p in 369 hepatocellular carcinoma (HCC) tumor samples versus 49 normal liver tissues. Data were obtained from TCGA and log2(TPM + 1) was used to present expression level. TPM, transcripts per million reads. **B** Expression of miR-103a-3p in a cohort of HCC patients with different cancer stages. **C** Overall survival was compared between patients with HCC with a high expression level of miR-103a and those with a low level of miR-103a. **D** RT-qPCR analysis of miR-103a-3p levels in L02 cells and HCC cell lines. ***P < 0.001 *vs* L02 group. **E**–**L** Hccl-M3 and QGY-7703 cells were transfected with miR-103a-3p mimics or mimics control, miR-103a-3p inhibitor or inhibitor control, and then subjected to colony formation assays (**E**–**K**) or CCK-8 assay (**I**–**L**). Data are presented as means ± SD of three independent experiments. **P < 0.01, ***P < 0.001

To further verify the expression of miR-103a-3p in HCC, we collected liver hepatocellular carcinoma data from TCGA public datasets and found that the expression level of miR-103a-3p was markedly increased in primary HCC tissues compared with normal tissues (*P* < 0.001, Fig. 3A), and with the progression of cancer, stage II–III tumors showed progressively higher expression of miR-103a-3p than stage I tumors in the TCGA cohort (*P* < 0.001, Fig. 3B). The prognostic value of miR-103a-3p for liver cancer, evaluated using an online database for prognostic analysis (Kaplan‒Meier Plotter, www.kmplot.com), indicated that high miR-103a-3p expression in patients with liver cancer was positively correlated with poor overall survival (*P* = 0.02, Fig. 3C). These results indicated that the expression of miR-103a-3p was upregulated in HCC and that increased expression of miR-103a-3p might serve as a prognostic factor for poor outcome in patients with HCC.

### miR-103a-3p promotes the proliferation, migration and invasion of HCC cells

**Fig. 4:**
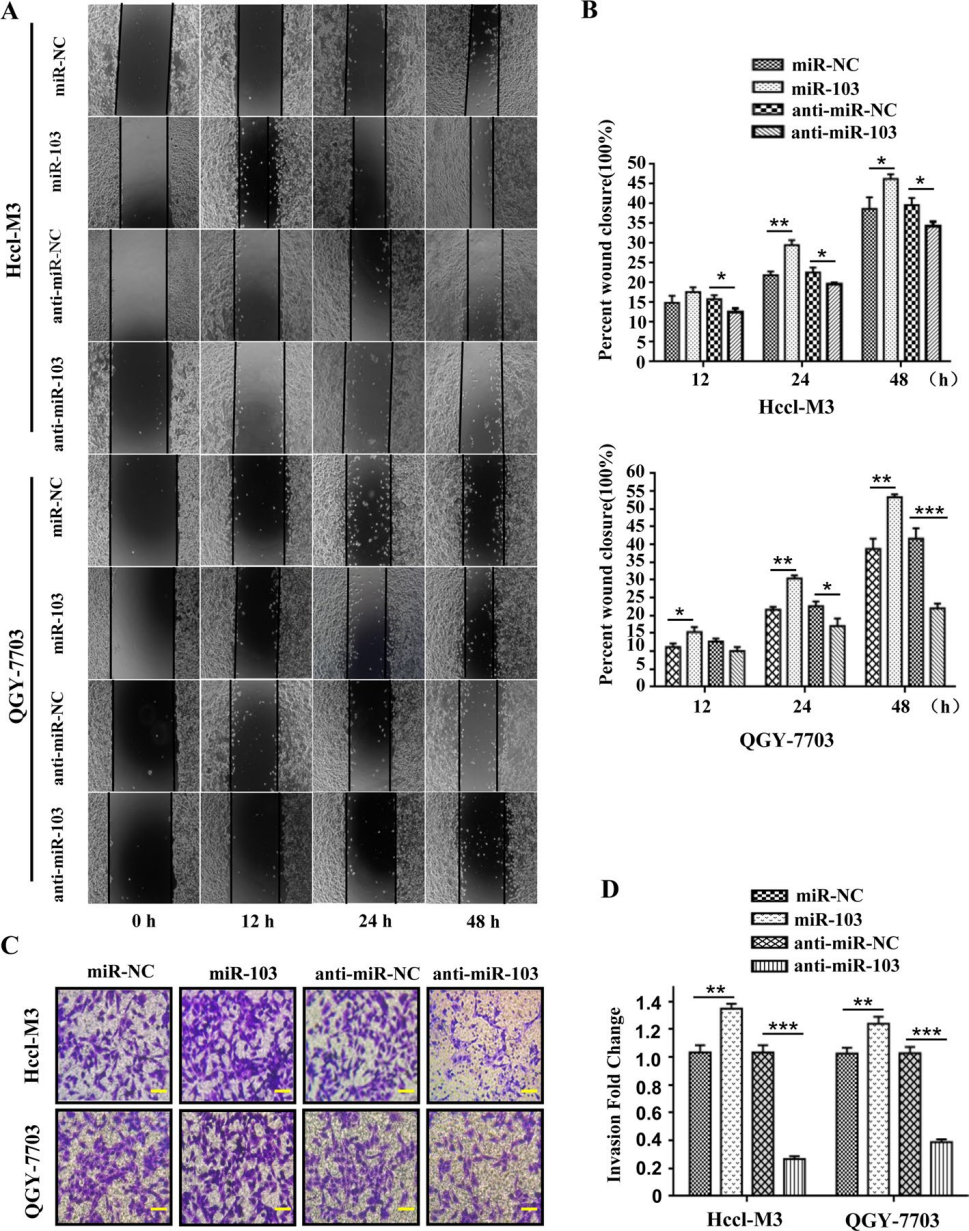
miR-103a-3p promotes HCC cells migration and invasion. Hccl-M3 and QGY-7703 cells were transfected with miR-103a-3p mimics, mimics control, miR-103a-3p inhibitor or inhibitor control, 24 h later, the migratory capacity was evaluated by wound healing assay (**A**, **B**) and the invasive ability was detected by transwell matrigel invasion assay (**C**, **D**). Scale bars, 50 μm. Data are shown as means ± SD from three independent experiments. *P < 0.05, **P < 0.01, ***P < 0.001

To explore the biological function of miR-103a-3p in HCC cells, we measured the expression levels of miR-103a-3p in HCC cell lines. The RT‒qPCR results showed significantly higher expression of miR-103a-3p in the HCC cell lines PLC-PRF5, QGY-7703 and Hccl-M3 than in the normal human liver cell line L02 (*P* < 0.001, Fig. 3D), which was opposite to the results for EVA1A expression in the HCC cell lines (Fig. 1E). Colony formation assays were performed to assess the roles of miR-103a-3p in the proliferation of HCC cells. The results revealed that overexpression of miR-103a-3p significantly promoted colony formation of Hccl-M3 cells and QGY-7703 cells (*P* < 0.01, Fig. 3E, F). In contrast, colony formation of HCC cells transfected with miR-103a-3p inhibitor was significantly suppressed (*P* < 0.01, Fig. 3G, H). CCK-8 assays also showed that upregulation of miR-103a-3p promoted the proliferation of Hccl-M3 cells and QGY-7703 cells and that downregulation of miR-103a-3p inhibited the proliferation of HCC cells (*P* < 0.01, Fig. 3I–L). In addition, we used wound healing and transwell assays to explore the effects of miR-103a-3p on the migration and invasion of Hccl-M3 and QGY-7703 cells. The results of the wound healing assay showed that the wound areas of cells transfected with miR-103a-3p mimics were significantly smaller than those of the control group, especially at 24 h (*P* < 0.01, Fig. 4A, B) and 48 h (*P* < 0.05, Fig. 4A, B) after transfection, while those transfected with miR-103a-3p inhibitors showed lower migratory capacities than the control group (*P* < 0.05, Fig. 4A, B). The results of the invasion assay demonstrated that the neoplasm invasiveness of HCC cells after transfection with miR-103a-3p mimics was significantly improved (*P* < 0.01, Fig. 4C, D), and those transfected with miR-103a-3p inhibitors showed lower invasiveness (*P* < 0.001, Fig. 4C, D). Together, these results demonstrated that miR-103a-3p promotes the proliferation, migration and invasion of HCC cells and implied that miR-103a-3p functions as a pro-oncogenic miRNA in HCC.

### Downregulation of EVA1A is essential for miR-103a-3p-mediated cancer-promoting effects in HCC cells

**Fig. 5:**
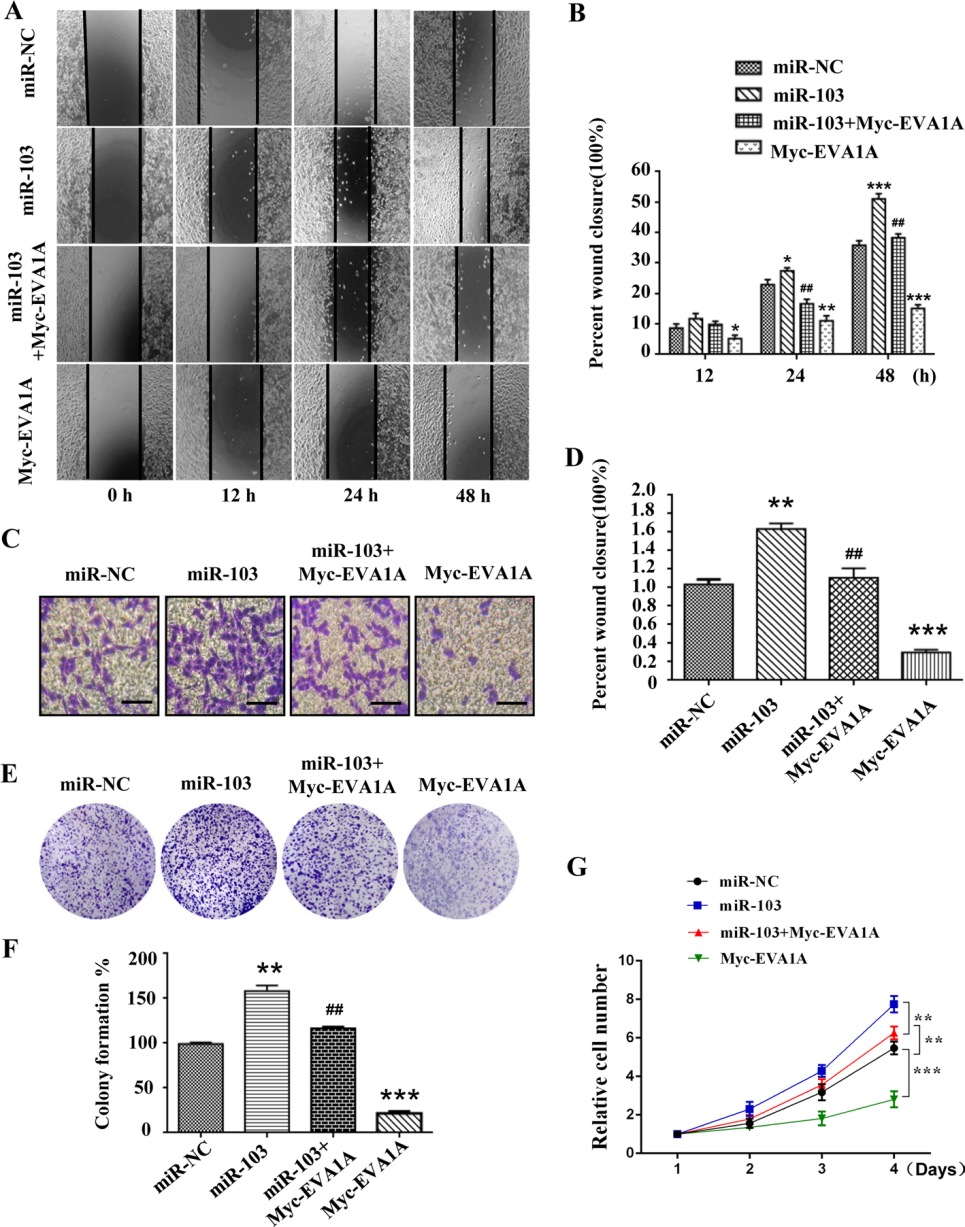
EVA1A attenuates the cancer-promoting effects of miR-103a-3p in HCC cells. Hccl-M3 cells were transfected with miR-103a-3p mimics or co-transfected with miR-103a-3p mimics and Myc-EVA1A plasmid, 24 h later, the migratory capacity was evaluated by wound healing assay (**A**, **B**), the invasive ability was detected by transwell matrigel invasion assay (**C**, **D**), and the proliferative capacity was analyzed based on colony formation (**E**, **F**) and CCK-8 assay (**G**). Scale bars, 50 μm. Data are shown as means ± SD from three independent experiments. *P < 0.05, **P < 0.01, ***P < 0.001 vs miR-NC group, ^##^*P* < 0.01 vs miR-103 group

We have reported that EVA1A is a potential tumor suppressor gene for HCC and that its downregulation is associated with poor clinical outcomes for HCC patients [10], so we evaluated the effect of EVA1A on the malignant action of miR-103a-3p in HCC. We conducted wound healing, transwell, colony formation and CCK-8 assays in Hccl-M3 cells. As the results showed, there was an evident decrease in migration, invasion and proliferation activity in the group co-transfected with miR-103a-3p and Myc-EVA1A plasmids compared with the group transfected with miR-103a-3p overexpression alone (*P* < 0.01, Fig. 5). Upregulation of miR-103a-3p significantly promoted the migration, invasion and proliferation of Hccl-M3 cells (*P* < 0.01, Fig. 5), and overexpression of EVA1A significantly suppressed the migration, invasion and proliferation of Hccl-M3 cells (*P* < 0.001, Fig. 5), indicating that EVA1A attenuates the cancer-promoting roles that miR-103a-3p plays in Hccl-M3 cells. Taken together, these results suggest that EVA1A is a bona fide target of miR-103a-3p and that its downregulation is involved in the tumor-promoting action of miR-103a-3p in HCC.

The epithelial-mesenchymal transition (EMT) is the critical factor for cancer cell metastasis [25, 26]. Recently, we have reported that EVA1A could inhibit EMT in HCC cells, by upregulating epithelial marker E-cadherin levels and downregulating mesenchymal markers N-cadherin and Vimen levels [10]. In the present study, we further investigated the molecular mechanisms involved. Studies have shown that abnormal activation of JAK/STAT3(Janus kinase/signal transducer and activator of transcription 3) signaling pathway can promote EMT and promote HCC progression [27–30], so we detected this pathway and found that the phospho-JAK2 levels, the phospho-STAT3 levels and the downstream gene MMP9 (matrix metallopeptidase 9) levels were significantly increased in miR-103a-3p overexpressing cells, while all of these protein levels dropped dramatically in EVA1A overexpressing cells, and the activation of JAK2/STAT3 signaling pathway was significantly attenuated in cells co-expressing EVA1A and miR-103a-3p compared with cells overexpressing miR-103a-3p alone (Additional file 1: Fig. S1). These data suggest that the pro-metastatic effect of miR-103a-3p in HCC may be mediated by EMT after JAK2/STAT3 signaling pathway activation by downregulating EVA1A.

Furthermore, the correlation of the expression of EVA1A with the clinicopathological features of HCC patients was analyzed by HCC tissue microarray. According to the EVA1A IHC staining score, samples were divided into high and low expression group. Results showed that a low EVA1A expression was related to TNM stage (*P* = 0.032), tumor size (*P* = 0.0161), lymph node metastasis (*P* = 0.0168) and distant metastasis (*P* = 0.0185) (Additional file 1: Table S1). The associations of EVA1A expression with gender and age were not significant (Additional file 1: Table S1). The results indicate that EVA1A might be correlated with the development and progression of HCC and its low expression might be a potential biomarker for HCC metastasis.

### Overexpression of EVA1A does not affect G2/M transition or autophagy level in Hccl-M3 cells

**Fig. 6:**
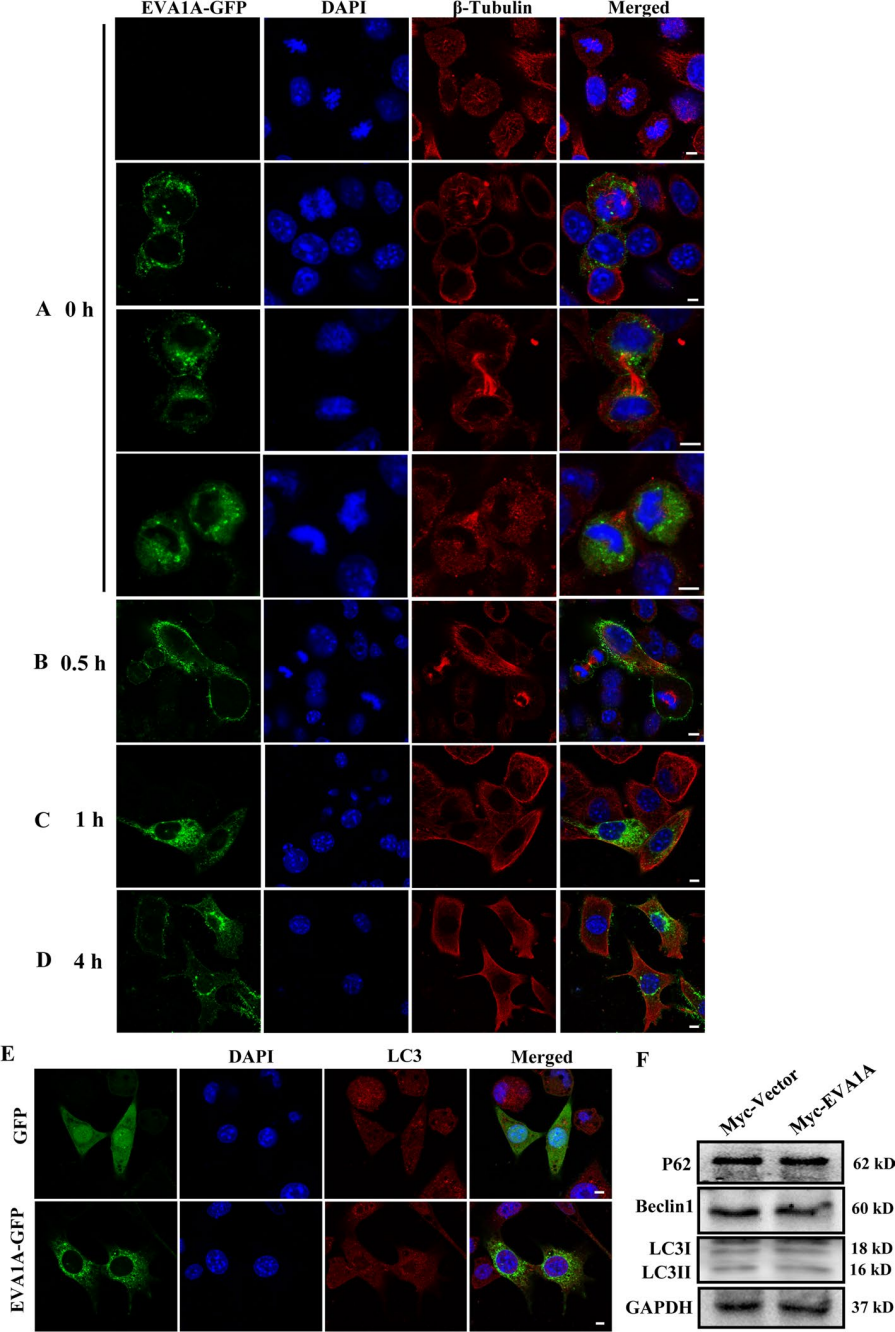
Effect of overexpression EVA1A on mitotic progression and autophagy. Hccl-M3 cells transfected with EVA1A-GFP plasmid were synchronized at mitotic phase by 100 ng/ml nocodazole treatment for 14 h. 0 h (**A**), 0.5 h (**B**), 1 h (**C**) and 4 h (**D**) after removal of nocodazole, cells were fixed, stained with anti-β-Tubulin and DAPI, and imaged by confocal microscopy. Scale bars, 5 μm. (**E**) Hccl-M3 cells were transfected with GFP empty vector or EVA1A-GFP plasmid, 24 h later, cells were fixed, stained with anti-LC3 and DAPI, and imaged by confocal microscopy. Scale bars, 5 μm. (**F**) Hccl-M3 cells were transfected with Myc-tag vector or Myc-EVA1A plasmid, 24 h later, cells were lysed and subjected to western blot

We next investigated how EVA1A inhibits HCC cell proliferation. One previous study has reported that ectopic expression of EVA1A in HepG2 cells caused cell death during G2/M transition by microtubule catastrophe, resulting a G2 peak decline [31]. However, we didn't find changes in G2 phase cells proportion upon EVA1A overexpression, and our previous results showed an evident increase in G0/G1 phase proportion and an evident decrease in S phase proportion in EVA1A overexpressing Hccl-M3 and OGY-7703 cells [10], which was consistent with the study of Shen Xue et al. [7]. To determine whether EVA1A overexpression affects G2/M transition or mitosis progression, Hccl-M3 cells overexpressing EVA1A-GFP were synchronized to M phase with nocodazole, a chemical which could bind to β-tubulin in microtubules to interfere with microtubule dynamics and inhibit mitotic spindle function, inducing cell arrest in G2/M phase [32], and released from M phase by removal of nocodazole for different time to observe the mitosis and spindle state of the cells. As shown in Fig. 6A, after nocodazole treatment for 14 h, microtubules were partially depolymerized and many cells arrested in mitosis. Notably, cells overexpressing EVA1A were also able to enter mitosis, such as entering telophase and cytokinesis (Fig. 6A), and chromosomes were being pulled toward the poles by the spindle (Fig. 6A, second panel), suggesting that EVA1A overexpression does not affect microtubule assembly to form the correct spindle, nor does it affect mitosis. 0.5 h after removal of nocodazole, microtubules began to reassemble, and the cells overexpressing EVA1A had significantly enhanced signals in the contractile ring during cytokinesis and the spindle at metaphase (Fig. 6B). 1 h and 4 h after removal of nocodazole, microtubule assembly was basically restored, and cells overexpressing EVA1A completed mitosis and entered interphase (Fig. 6C, D). These results indicates that EVA1A has no effect on microtubule assembly and EVA1A overexpressing cells could enter and complete mitosis normally.

Given the negative regulation of EVA1A by long antisense noncoding RNA, EVA1A-AS in HepG2 cells [31], we also checked the expression of EVA1A-AS in our cell system. In contrast to their findings, EVA1A-AS was also expressed in immortalized normal human liver cell line L02, and although the levels of EVA1A-AS in Huh7 and HepG2 cells were significantly higher than that in L02, the levels of EVA1A-AS in Hccl-M3 cells was almost the same as that in L02 cells (Additional file 1: Fig. S2), indicating that the suppressing effect of EVA1A-AS on EVA1A expression may be limited to some HCC cell lines. In addition, we also did not find the phenotype of lipid droplet accumulation in EVA1A overexpressing Hccl-M3 cells (Additional file 1: Fig. S3), suggesting that lipid droplet accumulation induce by depletion of EVA1A-AS in HepG2 cells is independent of EVA1A.

Considering that EVA1A is an important autophagy regulator [8], and studies have shown that EVA1A inhibits GBM cell and other cancer cell growth by inducing autophagy [5, 7], so we examined the effect of overexpression EVA1A on autophagy activity in Hccl-M3 cells by measuring the LC3 isoform B (LC3B) autophagy marker. Unexpectedly, cells overexpressing EVA1A-GFP did not show a significant increase in endogenous LC3B puncta compared with control cells overexpressing GFP (Fig. 6E), and analysis by western blot showed similar effects, the autophagy membrane protein LC3-II/I levels, the autophagy initiation molecule Beclin1 levels and the autophagic substrate protein p62 levels showed little difference between Myc-EVA1A overexpressing cells and control cells (Fig. 6F), suggesting that overexpression of EVA1A could not induce autophagy in Hccl-M3 cells.

### Inhibition of miR-103a-3p induces HCC cell apoptosis by upregulating the EVA1A-TP53 pathway

**Fig. 7:**
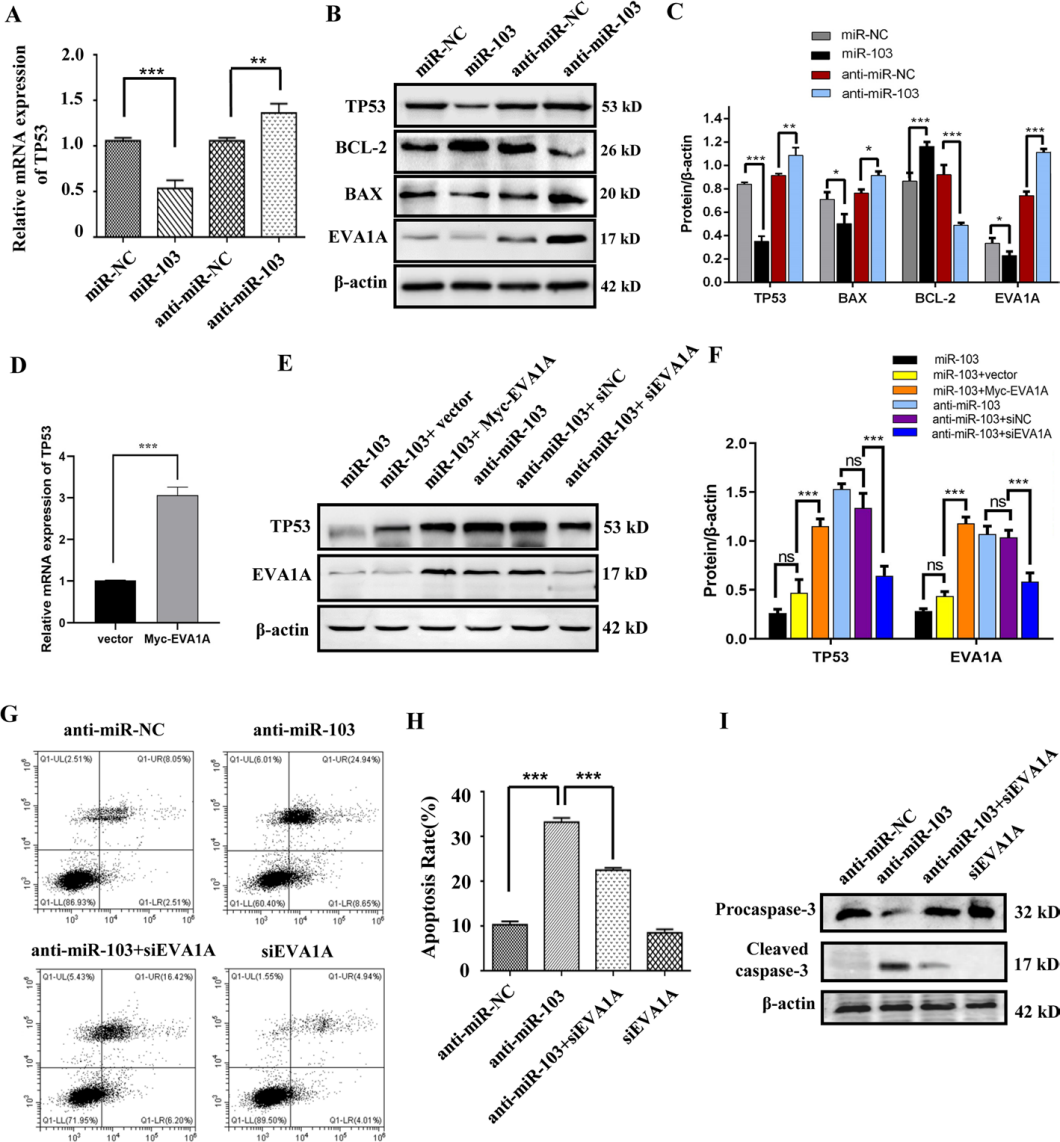
Repression of miR-103a-3p induces HCC cells apoptosis through upregulating EVA1A/TP53 pathway. **A** Hccl-M3 cells were transfected with miR-103a-3p mimics, mimics control, miR-103a-3p inhibitor or inhibitor control. 72 h after transfection, the TP53 mRNA levels was detected by RT-qPCR. **B** The protein levels of TP53, BCL-2, BAX and EVA1A was detected by western blot. **C** Quantitative analysis of the blot bands of TP53, BCL-2, BAX and EVA1A. **D** RT-qPCR analysis of TP53 mRNA in Hccl-M3 cells overexpressing EVA1A. **E** Hccl-M3 cells were transfected with miR-103a-3p mimics alone or together with Myc-EVA1A plasmid, or transfected with miR-103a-3p inhibitors alone or together with siRNA of EVA1A.Then the expression of TP53 and EVA1A was detected by western blot. **F** Quantitative analysis of the blot bands of TP53 and EVA1A. **G** Hccl-M3 cells were transfected with miR-103a-3p inhibitor or siRNA of EVA1A, or both of them, then the apoptosis rate was assessed by Annexin V-FITC/PI staining and flow cytometry assay. **H** Statistical apoptosis rate of each group. Data are shown as means ± SD from three independent experiments. **P < 0.01, ***P < 0.001. **I** Western blot analysis of caspase-3 activation

TP53 is an important anti-oncogene, and we previously reported that overexpression of EVA1A inhibits HCC cell proliferation by inducing apoptosis by upregulating TP53 [10]. Therefore, we further explored whether the anticancer effect of downregulating miR-103a-3p is related to apoptosis and whether EVA1A and TP53 are involved in this process. RT‒qPCR results showed that TP53 mRNA levels were significantly upregulated (*P* < 0.01) upon miR-103a-3p inhibition but significantly downregulated (*P* < 0.001) upon miR-103a-3p overexpression (Fig. 7A). Western blot analysis showed that the levels of TP53, BAX and EVA1A were significantly increased and the level of BCL-2 was dramatically decreased in miR-103a-3p-inhibited cells, while miR-103a-3p overexpression led to the opposite results (Fig. 7B, C), indicating that miR-103a-3p suppresses TP53/BAX-mediated apoptosis and that inhibition of miR-103a-3p promotes TP53/BAX-mediated apoptosis, as shown in the cell apoptosis profiles obtained by flow cytometry (*P* < 0.001, Fig. 7G, H) and caspase-3 activation by western blot (Fig. 7I). Importantly, co-expressing exogenous EVA1A evidently reversed the inhibitory effects of miR-103a-3p on TP53 expression, and knockdown of EVA1A greatly weakened the enhancement of miR-103a-3p inhibition on TP53 expression (*P* < 0.001, Fig. 7E, F). In addition, overexpressing EVA1A directly upregulated the mRNA level of TP53 (*P* < 0.001, Fig. 7D), which was consistent with our previous finding that overexpressing EVA1A upregulated the protein level of TP53 [10]. These results suggested that the negative regulation of TP53 expression by miR-103a-3p is primarily mediated by EVA1A. Consequently, knockdown of EVA1A significantly reduced cell apoptosis caused by miR-103a-3p inhibition (*P* < 0.001, Fig. 7G–I). Together, these results suggest that the cell apoptosis achieved by miR-103a-3p inhibition is dependent on upregulating the EVA1A/TP53 pathway and that miR-103a-3p potentially functions as a pro-oncogenic miRNA by targeting EVA1A and thereby inhibiting apoptosis and enhancing proliferation.

### Inhibition of miR-103a-3p causes mitochondrial dysfunction dependent of EVA1A upregulation

**Fig. 8:**
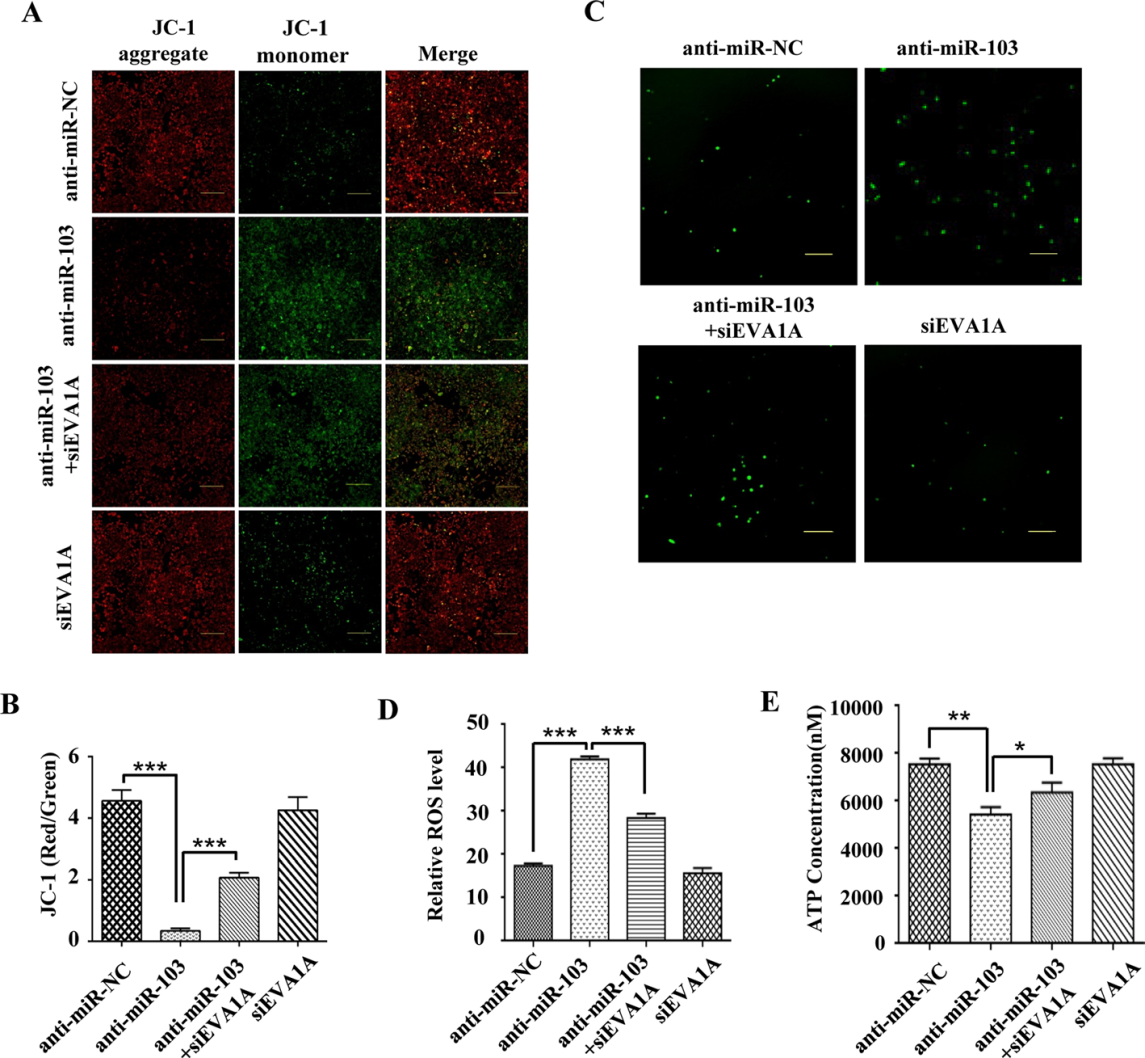
Inhibition of miR-103a-3p triggers EVA1A-dependent mitochondrial dysfunction. **A**, **B** Hccl-M3 cells were transfected with miR-103a-3p inhibitor or siRNA of EVA1A, or both of them. The changes of mitochondrial membrane potential were evaluated by JC-1 staining. **C**, **D** Intracellular ROS levels were determined by DCFH-DA probes. Scale bars, 200 μm. **E** Intracellular ATP levels were detected by the ATP Assay Kit. Data are presented as means ± SD from three independent experiments. *P < 0.05, **P < 0.01, ***P < 0.001

To further explore how inhibition of miR-103a-3p or upregulation of EVA1A affects cell apoptosis, we evaluated the mitochondrial membrane potential (MMP), intracellular ROS level and ATP level. In healthy cells, the JC-1 dye aggregates in the mitochondria and emits red fluorescence. When the MMP is lost, it depolymerizes into monomers and is released from the mitochondria into the cytoplasm, emitting green fluorescence. The results showed that inhibition of miR-103a-3p caused a significant enhancement in green fluorescence and a clear drop in red fluorescence (*P* < 0.001, Fig. 8A, B), indicating that inhibition of miR-103a-3p led to a significant decline in MMP, which was consistent with the finding that overexpressing EVA1A significantly reduced the MMP of HCC cells [10]. Not surprisingly, EVA1A knockdown greatly rescued the decline in MMP caused by miR-103a-3p inhibition, and EVA1A knockdown itself had almost no effect on MMP (*P* ≥ 0.05, Fig. 8A, B). In addition, inhibition of miR-103a-3p caused a significant increase in ROS production (*P* < 0.001, Fig. 8C, D) and a clear decrease in ATP production (*P* < 0.01, Fig. 8E), which suggested that it caused mitochondrial dysfunction. Likewise, EVA1A knockdown attenuated the changes in ROS and ATP levels induced by miR-103a-3p inhibition (Fig. 8C–E), while it had little effect on the production of ROS and ATP per se (*P* ≥ 0.05, Fig. 8C–E). In summary, inhibition of miR-103a-3p reduced cell MMP, promoted ROS production and reduced ATP production, and simultaneous knockdown of EVA1A reduced these effects, which revealed that the cell apoptosis induced by miR-103a-3p inhibition originates from mitochondrial dysfunction dependent of EVA1A upregulation.

## Discussion

**Fig. 9:**
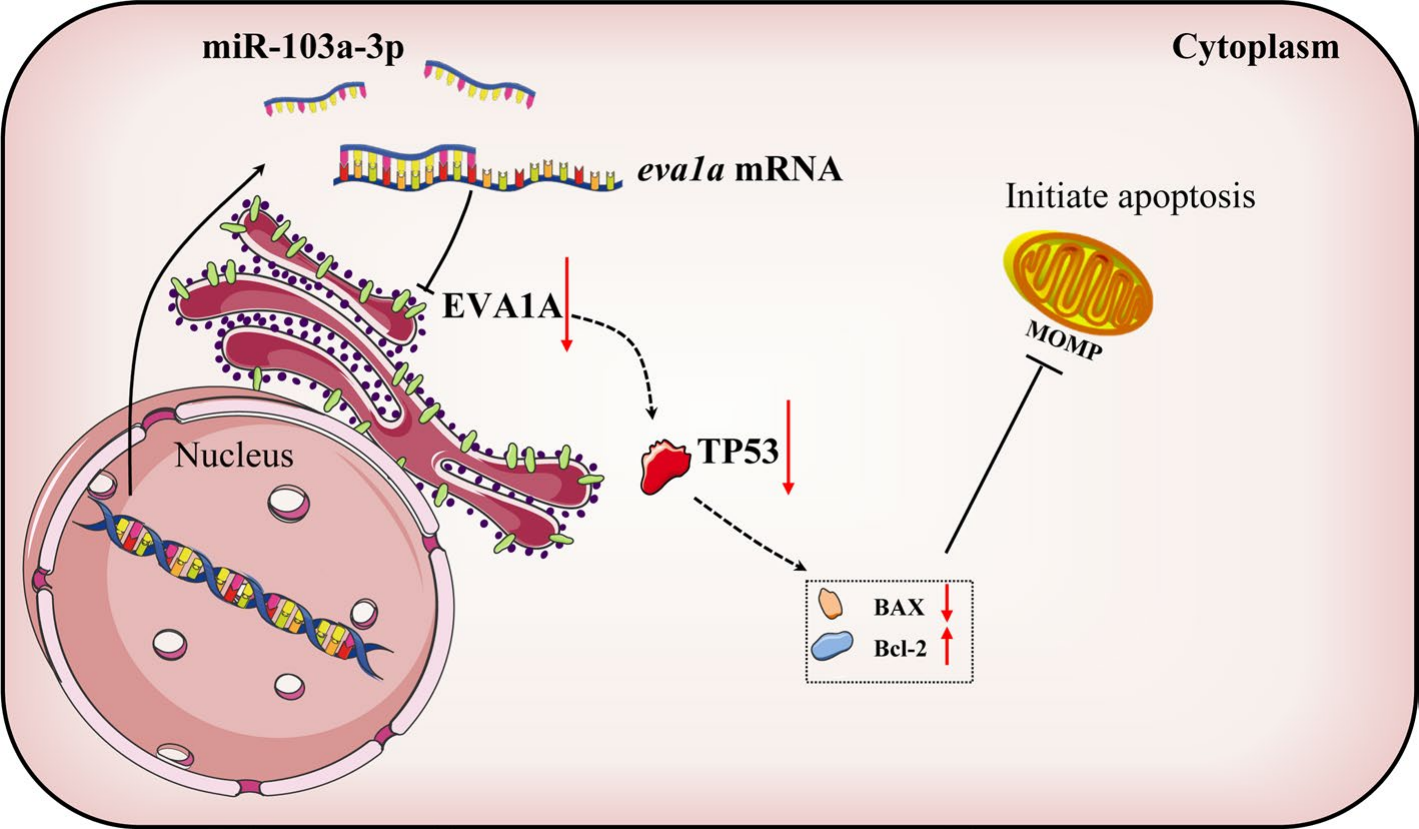
Schematic representation of a model depicting the underlying molecular mechanisms of the miR-103a-3p /EVA1A/TP53 regulatory axis in HCC. The increased expression of miR-103a-3p of human hepatocyte targets EVA1A mRNA and inhibits its expression, resulting in a decline of TP53 and BAX (pro-apoptotic protein), an increase of BCL-2(anti-apoptotic protein), and repression of MOMP, thereby inhibiting cell apoptosis and causing the tumor suppressor effect of EVA1A to be weakened. MOMP, mitochondrial outer membrane permeabilization

EVA1A, known for its autophagy regulation function, plays an important role in numerous physiological and pathological processes [33]. Recently, the downregulation of EVA1A and its tumor suppressor activity was shown to be a high profile. It has been found that EVA1A is significantly downregulated in human tumor tissues such as liver cancer, esophageal squamous cell carcinoma, gastric adenocarcinoma and pancreatic tumors [9, 10, 34], indicating that it may be involved in the occurrence or development of these tumors. Overexpression of EVA1A can inhibit tumor cell proliferation by inducing apoptosis in cervical cancer HeLa cells [3], non-small cell lung cancer H1299 cells [6], and glioblastoma SHG44, U87, and U251 cell lines [7]. Moreover, our recent research found that EVA1A can inhibit HCC cell migration, invasion, and proliferation and induce cell apoptosis and cell cycle arrest by upregulating TP53 [10]. In this study, we show for the first time that the tumor suppressive activity of EVA1A in HCC cells can be inhibited by miR-103a-3p. miR-103a-3p can target and negatively regulate EVA1A, which illuminates the regulatory mechanism of miR-103a-3p to promote HCC. We also reveal that miR-103a-3p promotes HCC cell growth and mobility by targeting EVA1A and further downregulating TP53; in turn, downregulation of miR-103a-3p induces HCC cell apoptosis by upregulating the EVA1A/TP53 pathway (Fig. 9).

As a member of the miR-103/107 family, miR-103 shows abnormal expression in a variety of cancers and might act as an oncogene or tumor suppressor gene in different cancer types. For instance, miR-103 is upregulated in numerous types of cancers, including neuroblastoma [14], gastric cancer [15], breast cancer [16] and colorectal cancer [17], where it functions as an oncogene, but it is downregulated in non-small cell lung cancer, where it functions as a tumor suppressor gene [13]. Additionally, miR-103a-3p suppresses cell proliferation and invasion by targeting tumor protein D52 in prostate cancer [18] but promotes human gastric cancer cell proliferation by targeting activating transcription factor 7 (ATF7) [19], and it also promotes tumor growth and glycolysis through the Hippo pathway in colorectal cancer [20]. Moreover, miR-103a-3p regulates proliferation and apoptosis by targeting RCAN1 in oral squamous cell carcinoma (OSCC) cells [21], miR-103 promotes HCC growth by inhibiting AKAP12 [35] or by promoting glucose metabolism function [27], and miR-103 promotes metastasis and EMT by inhibiting LATS2 [36]. Thus, miR-103 usually targets different genes in different cancers or targets different genes in the same cancer but exerts different functions. To date, miR-103 has been identified as an oncogene in HCC; however, the relationship between miR-103a-3p and EVA1A in HCC remains unclear and merits further investigation.

Our study adds miR-103a-3p to the list of regulators of EVA1A, which previously included miR-125b and antisense lncRNA EVA1A-AS [11, 31]. This study is the first to comprehensively analyze the role and molecular mechanism of miR-103a-3p-EVA1A interplay in HCC growth and metastasis. In HCC tissues and cell lines, we confirmed that the expression of EVA1A is downregulated, and the expression of miR-103a-3p in HCC tissues and cells is upregulated, which is consistent with previous studies [35–37]. We also analyzed the correlation of miR-103a-3p expression with HCC tumor stages and its prognostic prediction value and found that high miR-103a-3p expression was positively correlated with poor overall survival, so miR-103a-3p could serve as a potential biomarker and a prognostic factor for poor outcome in patients with HCC. Then, we proved that EVA1A is targeted by miR-103a-3p and downregulated in HCC cells. EVA1A upregulation attenuates the cancer-promoting effects of miR-103a-3p, and EVA1A downregulation greatly reduces cell apoptosis induced by miR-103a-3p inhibition. Our study adds weight to the notion that EVA1A functions as a tumor suppressor in hepatocytes. Furthermore, our data show that by regulating EVA1A expression, miR-103a-3p plays an oncogenic role in hepatocarcinogenesis.

Combining our previous study [10], we found that TP53 could be positively regulated by EVA1A and negatively regulated by miR-103a-3p at both the mRNA and protein levels, and the negative regulation of TP53 expression by miR-103a-3p is primarily mediated by EVA1A. It is difficult to rule out that miR-103a-3p could also target TP53 and directly regulate its expression. If so, miR-103a-3p may act synergistically with EVA1A to regulate TP53 expression. Moreover, the mechanism by which EVA1A upregulates TP53 is unclear. In this study, overexpressing EVA1A significantly enhanced TP53 mRNA levels, implying that it is a direct stress response. As an endoplasmic reticulum-located protein, a high EVA1A protein load may cause ER stress, which can cause an increase in TP53 [38], the details of which should be investigated in the future. TP53, as a crucial anticancer gene, controls cell apoptosis, the cell cycle, and cell invasion [39, 40], and it was also reported to inhibit EMT and metastasis by negatively regulating several EMT-inducing transcription factors and regulatory molecules [7]. Whether miR-103a-3p regulates EMT and metastasis through TP53 signaling in HCC remains to be confirmed.

Additionally, as we reported previously, EVA1A overexpression induces mitochondrial outer membrane permeabilization (MOMP), decreases MMP and initiates cell apoptosis [10]. Based on this, this study also explored the effect of miR-103a-3p on mitochondrial function, and the results showed that repression of miR-103a-3p can damage the normal physiological functions of mitochondria, causing a decrease in MMP and ATP production and an increase in ROS production, all of which are induction factors of apoptosis. Furthermore, we found that the cell apoptosis induced by miR-103a-3p inhibition depends on the EVA1A/TP53 pathway and that EVA1A also mediates the mitochondrial dysfunction caused by miR-103a-3p inhibition. One study with quantitative proteomics of EVA1A^−/−^ mouse brains showed that the proteins with altered expression are related to ATP synthesis, oxidative phosphorylation and the TCA cycle, implying that EVA1A may be involved in mitochondrial energy generation [41]. Since mitochondria play an important role in cell energy metabolism, reactive oxygen generation and cell apoptosis, whether EVA1A and miR-103a-3p also affect the related disease process through mitochondrial quality control needs further research.

## Conclusions

In summary, our study identifies miR-103a-3p as a potential oncogene and an inhibitor of EVA1A in HCC cells. We found that miR-103a-3p is upregulated in HCC tissues and that high expression of miR-103a is associated with poor patient prognosis. We also show that miR-103a-3p promotes HCC cell growth by downregulating TP53 and promotes HCC cell migration by upregulating JAK2/STAT3 pathway in an EVA1A-dependent manner, thereby inhibiting apoptosis and enhancing proliferation and metastasis. We also provided novel evidence that miR-103a-3p and EVA1A antagonistically regulate mitochondrial function. These findings provide a platform for investigating the signaling pathways in HCC that are mediated by EVA1A and modulated by miR-103a-3p, offering new insights into the miRNA regulatory network in the development of HCC. The identification of the miR-103a-3p/EVA1A/TP53 regulatory axis contributes to a better understanding of the molecular mechanisms of HCC progression. Therefore, our study reveals that the downregulation of EVA1A by miR-103a-3p may act as a key mediator in HCC progression. miR-103a-3p may represent a prospective target for both HCC diagnosis and therapy.

## Supplementary Information

**Additional file 1: Fig. S1.** Western blot analysis of JAK2/STAT3 activation. Hccl-M3 cells were transfected with miR-103a-3p mimics, Myc-EVA1A plasmid or co-transfected with both, 72 h after transfection, the protein levels of phospho-JAK2, phospho-STAT3 and MMP9 were detected by western blot. **Fig. S2.** The EVA1A-AS expression level in HCC cell lines. Total RNAs from L02, Hccl-M3, Huh7 and HepG2 cells were supplied for EVA1A-AS and GAPDH specific semi-quantitative RT-PCR. Three independent experiments were performed. **Fig. S3.** Effect of overexpression EVA1A on lipid droplet distribution in Hccl-M3 cells. (A) The transfection efficiency of TMEM166-GFP or GFP vector in Hccl-M3 cells. (B) Hccl-M3 cells were transfected with GFP empty vector or EVA1A-GFP plasmid, 24 h later, cells were applied for oil red O staining. Bars represent 10 μm. **Table S1.** The association between EVA1A expression and clinicopathologic features in HCC patients.

## Data Availability

Datasets on expression levels of miR-103a-3p from HCC patients were obtained from The Cancer Genome Atlas (TCGA; https://tcga-data.ci.nih.gov/tcga/), and were analyzed through UALCAN database (http://ualcan.path.uab.edu/).

## Abbreviations

HCC: Hepatocellular carcinoma
ATF7: Activating transcription factor 7
RCAN1: Calcineurin1
OSCC: Oral squamous cell carcinoma
VE-Cad: VE-Cadherin
EMT: Epithelial-mesenchymal transition
NOR1: Oxidored-nitro domain-containing protein 1
LATS2: Large tumor suppressor kinase 2
FOXA2: Forkhead box A2
EVA1A: Eva-1 homolog A
DCFH-DA: 2, 7-Dichlorofluorescein diacetate
TCGA: The Cancer Genome Atlas
NC: Navigate control
3′-UTR: 3′-Untranslated region
MMP: Mitochondrial membrane potential
ROS: Reactive oxygen species
MOMP: Mitochondrial outer membrane permeabilization
TPM: Transcripts per million reads
VEGF: Vascular endothelial growth factor
AKAP12: A kinase anchoring protein 12
EMT: Epithelial-mesenchymal transition
JAK: Janus kinase
STAT3: Signal transducer and activator of transcription 3
MMP9: Matrix metallopeptidase 9

## References

[1] Sung H, Ferlay J, Siegel RL, Laversanne M, Soerjomataram I, Jemal A, Bray F (2021). Global Cancer Statistics 2020: GLOBOCAN estimates of incidence and mortality worldwide for 36 cancers in 185 countries. CA Cancer J Clin.

[2] Zeng H, Zheng R, Guo Y, Zhang S, Zou X, Wang N, Zhang L, Tang J, Chen J, Wei K (2015). Cancer survival in China, 2003–2005: a population-based study. Int J Cancer.

[3] Wang L, Yu C, Lu Y, He P, Guo J, Zhang C, Song Q, Ma D, Shi T, Chen Y (2007). TMEM166, a novel transmembrane protein, regulates cell autophagy and apoptosis. Apoptosis.

[4] Li L, Khatibi NH, Hu Q, Yan J, Chen C, Han J, Ma D, Chen Y, Zhou C (2012). Transmembrane protein 166 regulates autophagic and apoptotic activities following focal cerebral ischemic injury in rats. Exp Neurol.

[5] Chang Y, Li Y, Hu J, Guo J, Xu D, Xie H, Lv X, Shi T, Chen Y (2013). Adenovirus vector-mediated expression of TMEM166 inhibits human cancer cell growth by autophagy and apoptosis in vitro and in vivo. Cancer Lett.

[6] Xie H, Hu J, Pan H, Lou Y, Lv P, Chen Y (2014). Adenovirus vector-mediated FAM176A overexpression induces cell death in human H1299 non-small cell lung cancer cells. BMB Rep.

[7] Shen X, Kan S, Liu Z, Lu G, Zhang X, Chen Y, Bai Y (2017). EVA1A inhibits GBM cell proliferation by inducing autophagy and apoptosis. Exp Cell Res.

[8] Hu J, Li G, Qu L, Li N, Liu W, Xia D, Hongdu B, Lin X, Xu C, Lou Y (2016). TMEM166/EVA1A interacts with ATG16L1 and induces autophagosome formation and cell death. Cell Death Dis.

[9] Xu D, Yang F, He H, Hu J, Lv X, Ma D, Chen YY (2013). Expression of TMEM166 protein in human normal and tumor tissues. Appl Immunohistochem Mol Morphol.

[10] Yang J, Wang B, Xu Q, Yang Y, Hou L, Yin K, Guo Q, Hua Y, Zhang L, Li Y (2021). TMEM166 inhibits cell proliferation, migration and invasion in hepatocellular carcinoma via upregulating TP53. Mol Cell Biochem.

[11] Ren WW, Li DD, Chen X, Li XL, He YP, Guo LH, Liu LN, Sun LP, Zhang XP (2018). MicroRNA-125b reverses oxaliplatin resistance in hepatocellular carcinoma by negatively regulating EVA1A mediated autophagy. Cell Death Dis.

[12] Fu Y, Lin L, Xia L (2019). MiR-107 function as a tumor suppressor gene in colorectal cancer by targeting transferrin receptor 1. Cell Mol Biol Lett.

[13] Yang D, Wang JJ, Li JS, Xu QY (2018). miR-103 Functions as a tumor suppressor by directly targeting programmed cell death 10 in NSCLC. Oncol Res.

[14] Annibali D, Gioia U, Savino M, Laneve P, Caffarelli E, Nasi S (2012). A new module in neural differentiation control: two microRNAs upregulated by retinoic acid, miR-9 and -103, target the differentiation inhibitor ID2. PLoS ONE.

[15] Zheng J, Liu Y, Qiao Y, Zhang L, Lu S (2017). miR-103 promotes proliferation and metastasis by targeting KLF4 in gastric cancer. Int J Mol Sci.

[16] Wang X, Wu X, Yan L, Shao J (2012). Serum miR-103 as a potential diagnostic biomarker for breast cancer. Nan Fang Yi Ke Da Xue Xue Bao.

[17] Nonaka R, Miyake Y, Hata T, Kagawa Y, Kato T, Osawa H, Nishimura J, Ikenaga M, Murata K, Uemura M (2015). Circulating miR-103 and miR-720 as novel serum biomarkers for patients with colorectal cancer. Int J Oncol.

[18] Ge J, Mao L, Xu W, Fang W, Wang N, Ye D, Dong Z, Guan H, Guan C (2021). miR-103a-3p suppresses cell proliferation and invasion by targeting tumor protein D52 in prostate cancer. J Invest Surg.

[19] Fang JH, Zhang ZJ, Shang LR, Luo YW, Lin YF, Yuan Y, Zhuang SM (2018). Hepatoma cell-secreted exosomal microRNA-103 increases vascular permeability and promotes metastasis by targeting junction proteins. Hepatology.

[20] Sun Z, Zhang Q, Yuan W, Li X, Chen C, Guo Y, Shao B, Dang Q, Zhou Q, Wang Q (2020). MiR-103a-3p promotes tumour glycolysis in colorectal cancer via hippo/YAP1/HIF1A axis. J Exp Clin Cancer Res.

[21] Zhang G, Chen Z, Zhang Y, Li T, Bao Y, Zhang S (2020). Inhibition of miR-103a-3p suppresses the proliferation in oral squamous cell carcinoma cells via targeting RCAN1. Neoplasma.

[22] Wu S, Zhao W, Sun M, He P, Lv H, Wang Q, Zhang S, Wu Q, Ling P, Chen S, Ma J (2022). Novel bi-layered dressing patches constructed with radially-oriented nanofibrous pattern and herbal compound-loaded hydrogel for accelerated diabetic wound healing. Appl Mater Today.

[23] Karakatsanis A, Papaconstantinou I, Gazouli M, Lyberopoulou A, Polymeneas G, Voros D (2013). Expression of microRNAs, miR-21, miR-31, miR-122, miR-145, miR-146a, miR-200c, miR-221, miR-222, and miR-223 in patients with hepatocellular carcinoma or intrahepatic cholangiocarcinoma and its prognostic significance. Mol Carcinog.

[24] Zhang Z, Zheng W, Hai J (2014). MicroRNA-148b expression is decreased in hepatocellular carcinoma and associated with prognosis. Med Oncol.

[25] Xu S, Zhang H, Wang A, Ma Y, Gan Y, Li G (2020). Silibinin suppresses epithelial-mesenchymal transition in human non-small cell lung cancer cells by restraining RHBDD1. Cell Mol Biol Lett.

[26] Ma S, Wei H, Wang C, Han J, Chen X, Li Y (2021). MiR-26b-5p inhibits cell proliferation and EMT by targeting MYCBP in triple-negative breast cancer. Cell Mol Biol Lett.

[27] Hin Tang JJ, Hao Thng DK, Lim JJ, Toh TB (2020). JAK/STAT signaling in hepatocellular carcinoma. Hepat Oncol..

[28] Sakamoto T, Kuboki S, Furukawa K, Takayashiki T, Takano S, Yoshizumi A, Ohtsuka M (2022). TRIM27-USP7 complex promotes tumour progression via STAT3 activation in human hepatocellular carcinoma. Liver Int..

[29] Lee C, Cheung ST (2019). STAT3: an emerging therapeutic target for hepatocellular carcinoma. Cancers (Basel)..

[30] Zhang C, Guo F, Xu G, Ma J, Shao F (2015). STAT3 cooperates with Twist to mediate epithelial-mesenchymal transition in human hepatocellular carcinoma cells. Oncol Rep.

[31] Niehus SE, Allister AB, Hoffmann A, Wiehlmann L, Tamura T, Tran DDH (2019). Myc/Max dependent intronic long antisense noncoding RNA, EVA1A-AS, suppresses the expression of Myc/Max dependent anti-proliferating gene EVA1A in a U2 dependent manner. Sci Rep.

[32] Mao L, Li N, Guo Y, Xu X, Gao L, Xu Y, Zhou L, Liu W (2013). AMPK phosphorylates GBF1 for mitotic Golgi disassembly. J Cell Sci.

[33] Zhao S, Wang H (2021). EVA1A plays an important role by regulating autophagy in physiological and pathological processes. Int J Mol Sci..

[34] Sun W, Ma XM, Bai JP, Zhang GQ, Zhu YJ, Ma HM, Guo H, Chen YY, Ding JB (2012). Transmembrane protein 166 expression in esophageal squamous cell carcinoma in Xinjiang, China. Asian Pac J Cancer Prev.

[35] Xia W, Ni J, Zhuang J, Qian L, Wang P, Wang J (2016). MiR-103 regulates hepatocellular carcinoma growth by targeting AKAP12. Int J Biochem Cell Biol.

[36] Han LL, Yin XR, Zhang SQ (2018). miR-103 promotes the metastasis and EMT of hepatocellular carcinoma by directly inhibiting LATS2. Int J Oncol.

[37] Liu Y, Zhang Y, Xiao B, Tang N, Hu J, Liang S, Pang Y, Xu H, Ao J, Yang J (2021). MiR-103a promotes tumour growth and influences glucose metabolism in hepatocellular carcinoma. Cell Death Dis.

[38] Lin WC, Chuang YC, Chang YS, Lai MD, Teng YN, Su IJ, Wang CC, Lee KH, Hung JH (2012). Endoplasmic reticulum stress stimulates p53 expression through NF-κB activation. PLoS ONE.

[39] Liebermann DA, Hoffman B, Vesely D (2007). p53 induced growth arrest versus apoptosis and its modulation by survival cytokines. Cell Cycle.

[40] Aubrey BJ, Kelly GL, Janic A, Herold MJ, Strasser A (2018). How does p53 induce apoptosis and how does this relate to p53-mediated tumour suppression?. Cell Death Differ.

[41] Li M, Lu G, Hu J, Shen X, Ju J, Gao Y, Qu L, Xia Y, Chen Y, Bai Y (2016). EVA1A/TMEM166 regulates embryonic neurogenesis by autophagy. Stem Cell Reports.

