# Supplementary material for: Down-regulation of EVA1A by miR-103a-3p promotes hepatocellular carcinoma cells proliferation and migration
Source: Cell Mol Biol Lett. 2022 Oct 22;27:93. doi: 10.1186/s11658-022-00388-8 (PMC9588234; doi:10.1186/s11658-022-00388-8)
Supplement: Supplementary file 1 — Additional file 1: Fig. S1. Western blot analysis of JAK2/STAT3 activation. Hccl-M3 cells were transfected with miR-103a-3p mimics, Myc-EVA1A plasmid or co-transfected with both, 72 h after transfection, the protein levels of phospho-JAK2, phospho-STAT3 and MMP9 were detected by western blot. Fig. S2. The EVA1A-AS expression level in HCC cell lines. Total RNAs from L02, Hccl-M3, Huh7 and HepG2 cells were supplied for EVA1A-AS and GAPDH specific semi-quantitative RT-PCR. Three independent experiments were performed. Fig. S3. Effect of overexpression EVA1A on lipid droplet distribution in Hccl-M3 cells. (A) The transfection efficiency of TMEM166-GFP or GFP vector in Hccl-M3 cells. (B) Hccl-M3 cells were transfected with GFP empty vector or EVA1A-GFP plasmid, 24 h later, cells were applied for oil red O staining. Bars represent 10 μm. Table S1. The association between EVA1A expression and clinicopathologic features in HCC patients. [file 11658_2022_388_MOESM1_ESM.pdf]

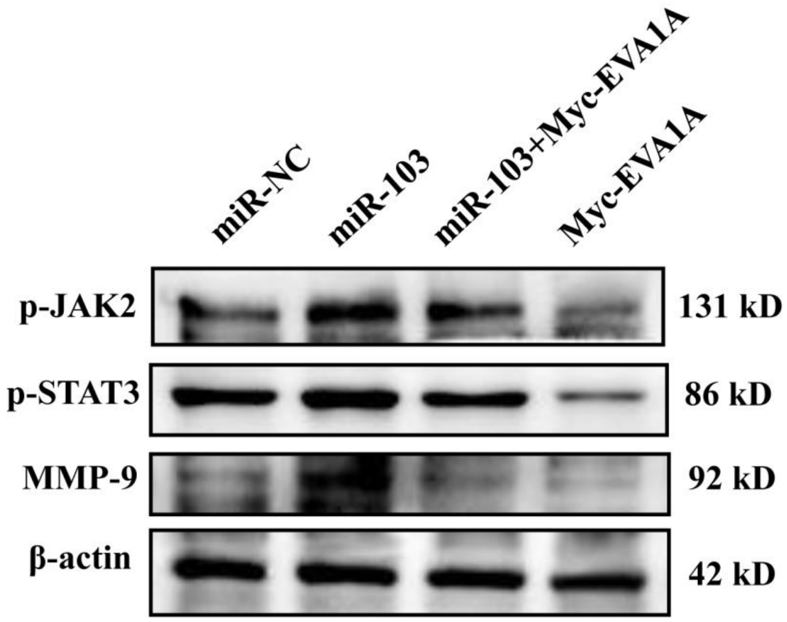

Fig. S1. Western blot analysis of JAK2/STAT3 activation. Hccl-M3 cells were transfected with miR-103a-3p mimics, Myc-EVA1A plasmid or co-transfected with both, 72 h after transfection, the protein levels of phospho-JAK2, phospho-STAT3 and MMP9 were detected by western blot.

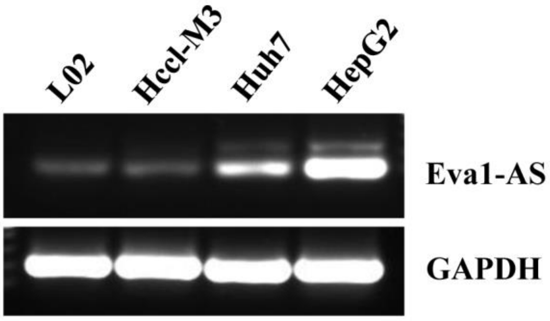

Fig. S2. The EVA1A-AS expression level in HCC cell lines. Total RNAs from L02, Hccl-M3, Huh7 and HepG2 cells were supplied for EVA1A-AS and GAPDH specific semi-quantitative RT-PCR. Three independent experiments were performed.

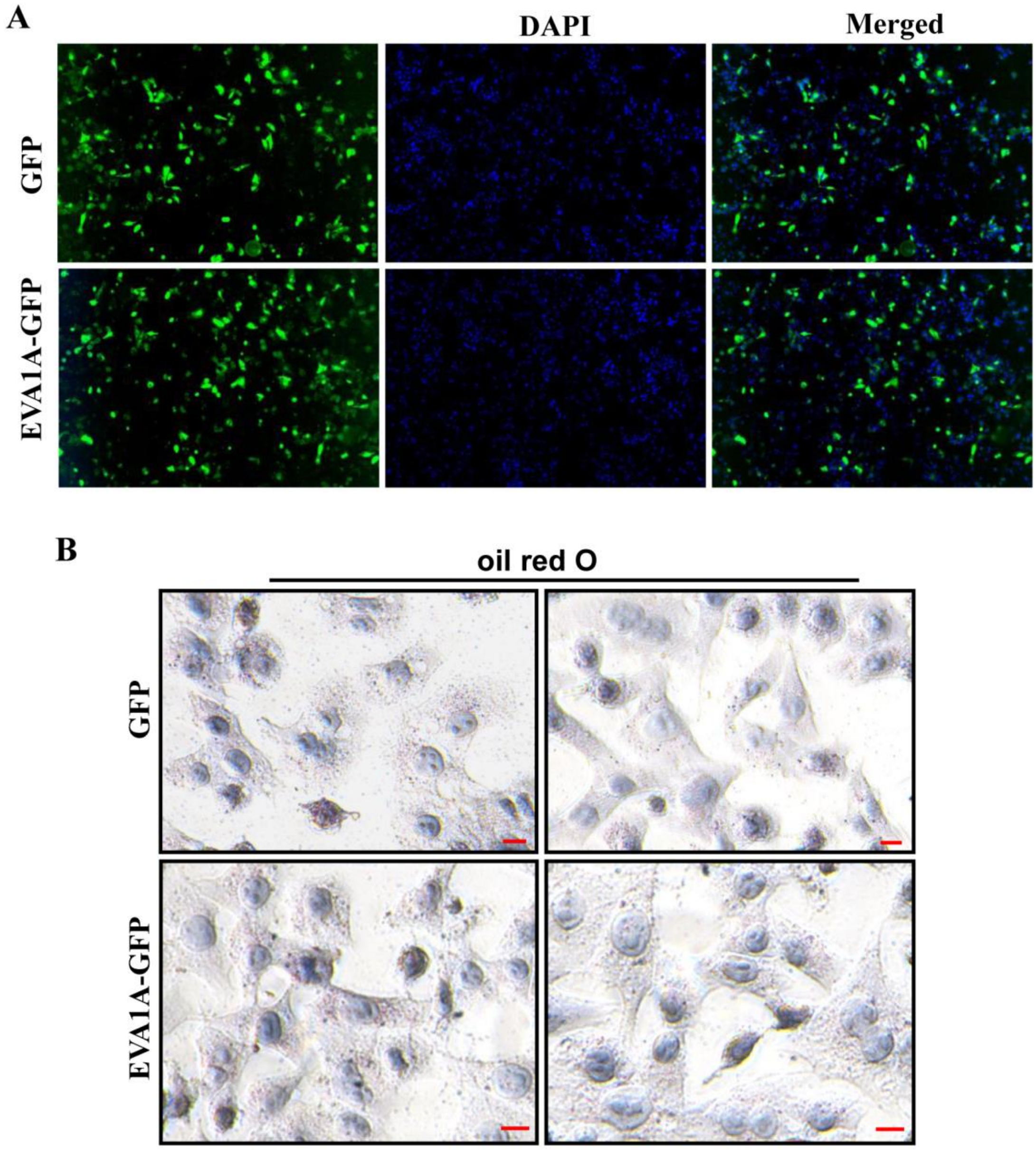

Fig. S3. Effect of overexpression EVA1A on lipid droplet distribution in Hccl-M3 cells. (A) The transfection efficiency of TMEM166-GFP or GFP vector in Hccl-M3 cells. (B) Hccl-M3 cells were transfected with GFP empty vector or EVA1A-GFP plasmid, 24 h later, cells were applied for oil red O staining. Bars represent 10 μm.

Table S1. The association between EVA1A expression and clinicopathologic features in HCC patients.

Clinicopathologicfeatures	EVA1A expression		X^2	*p*-value
	High(n=359)	Low(n=573)		
**Gender**			0.8239	0.3640
Male	241	368		
Female	118	205		
**Age**			0.1193	0.7298
≥50	209	327		
＜50	150	246		
**TNM stage**			4.597	0.0320*
I+II	174	319		
III+IV	185	254		
**Tumor size (cm)**			5.797	0.0161*
Large ≥5	197	268		
Small＜5	162	305		
**Lymph node metastasis**			5.722	0.0168*
Yes	5	24		
No	354	549		
**Distant metastases**			5.546	0.0185*
Yes	12	40		
No	347	533		

* Chip-square test, p-value < 0.05
